# Supplementary material for: Restricting SLC7A5-mediated Leucine uptake in T cells prevents acute GVHD and maintains GVT response
Source: EMBO Mol Med. 2025 May 21;17(7):1631–65. doi: 10.1038/s44321-025-00250-2 (PMC12254332; doi:10.1038/s44321-025-00250-2)
Supplement: Supplementary file 1 — Table EV1 [file 44321_2025_250_MOESM1_ESM.docx]

Table EV1. List of metabolites and inflammation parameters that are quantified in serum samples with Bruker IVDR software.

| Bruker's Software | Group | Variable | Unit |
| --- | --- | --- | --- |
| B.I.Quant-PS | Metabolites | 2-Aminobutyric acid | mmol/L |
| B.I.Quant-PS | Metabolites | 2-Hydroxybutyric acid | mmol/L |
| B.I.Quant-PS | Metabolites | 2-Oxoglutaric acid | mmol/L |
| B.I.Quant-PS | Metabolites | 3-Hydroxybutyric acid | mmol/L |
| B.I.Quant-PS | Metabolites | Acetic acid | mmol/L |
| B.I.Quant-PS | Metabolites | Acetoacetic acid | mmol/L |
| B.I.Quant-PS | Metabolites | Acetone | mmol/L |
| B.I.Quant-PS | Metabolites | Alanine | mmol/L |
| B.I.Quant-PS | Metabolites | Asparagine | mmol/L |
| B.I.Quant-PS | Metabolites | Choline | mmol/L |
| B.I.Quant-PS | Metabolites | Citric acid | mmol/L |
| B.I.Quant-PS | Metabolites | Creatine | mmol/L |
| B.I.Quant-PS | Metabolites | Creatinine | mmol/L |
| B.I.Quant-PS | Metabolites | D-Galactose | mmol/L |
| B.I.Quant-PS | Metabolites | Dimethylsulfone | mmol/L |
| B.I.Quant-PS | Metabolites | Ethanol | mmol/L |
| B.I.Quant-PS | Metabolites | Formic acid | mmol/L |
| B.I.Quant-PS | Metabolites | Glucose | mmol/L |
| B.I.Quant-PS | Metabolites | Glutamic acid | mmol/L |
| B.I.Quant-PS | Metabolites | Glutamine | mmol/L |
| B.I.Quant-PS | Metabolites | Glycerol | mmol/L |
| B.I.Quant-PS | Metabolites | Glycine | mmol/L |
| B.I.Quant-PS | Metabolites | Histidine | mmol/L |
| B.I.Quant-PS | Metabolites | Isoleucine | mmol/L |
| B.I.Quant-PS | Metabolites | Lactic acid | mmol/L |
| B.I.Quant-PS | Metabolites | Leucine | mmol/L |
| B.I.Quant-PS | Metabolites | Lysine | mmol/L |
| B.I.Quant-PS | Metabolites | Methionine | mmol/L |
| B.I.Quant-PS | Metabolites | N,N-Dimethylglycine | mmol/L |
| B.I.Quant-PS | Metabolites | Ornithine | mmol/L |
| B.I.Quant-PS | Metabolites | Phenylalanine | mmol/L |
| B.I.Quant-PS | Metabolites | Proline | mmol/L |
| B.I.Quant-PS | Metabolites | Pyruvic acid | mmol/L |
| B.I.Quant-PS | Metabolites | Sarcosine | mmol/L |
| B.I.Quant-PS | Metabolites | Succinic acid | mmol/L |
| B.I.Quant-PS | Metabolites | Threonine | mmol/L |
| B.I.Quant-PS | Metabolites | Trimethylamine-N-oxide | mmol/L |
| B.I.Quant-PS | Metabolites | Tyrosine | mmol/L |
| B.I.Quant-PS | Metabolites | Valine | mmol/L |
| PhenoRisk PACS RuO | Inflammation parameters | GlycA | p.d.u.^*^ |
| PhenoRisk PACS RuO | Inflammation parameters | GlycB | p.d.u.^*^ |
| PhenoRisk PACS RuO | Inflammation parameters | Glyc | p.d.u.^*^ |
| PhenoRisk PACS RuO | Inflammation parameters | SPC | p.d.u.^*^ |
| PhenoRisk PACS RuO | Inflammation parameters | Glyc/SPC | - |

*^*^p.d.u.: procedure defined units.*
