# Supplementary figures and images for: Restricting SLC7A5-mediated Leucine uptake in T cells prevents acute GVHD and maintains GVT response
Source: EMBO Mol Med. 2025 May 21;17(7):1631–65. doi: 10.1038/s44321-025-00250-2 (PMC12254332; doi:10.1038/s44321-025-00250-2)

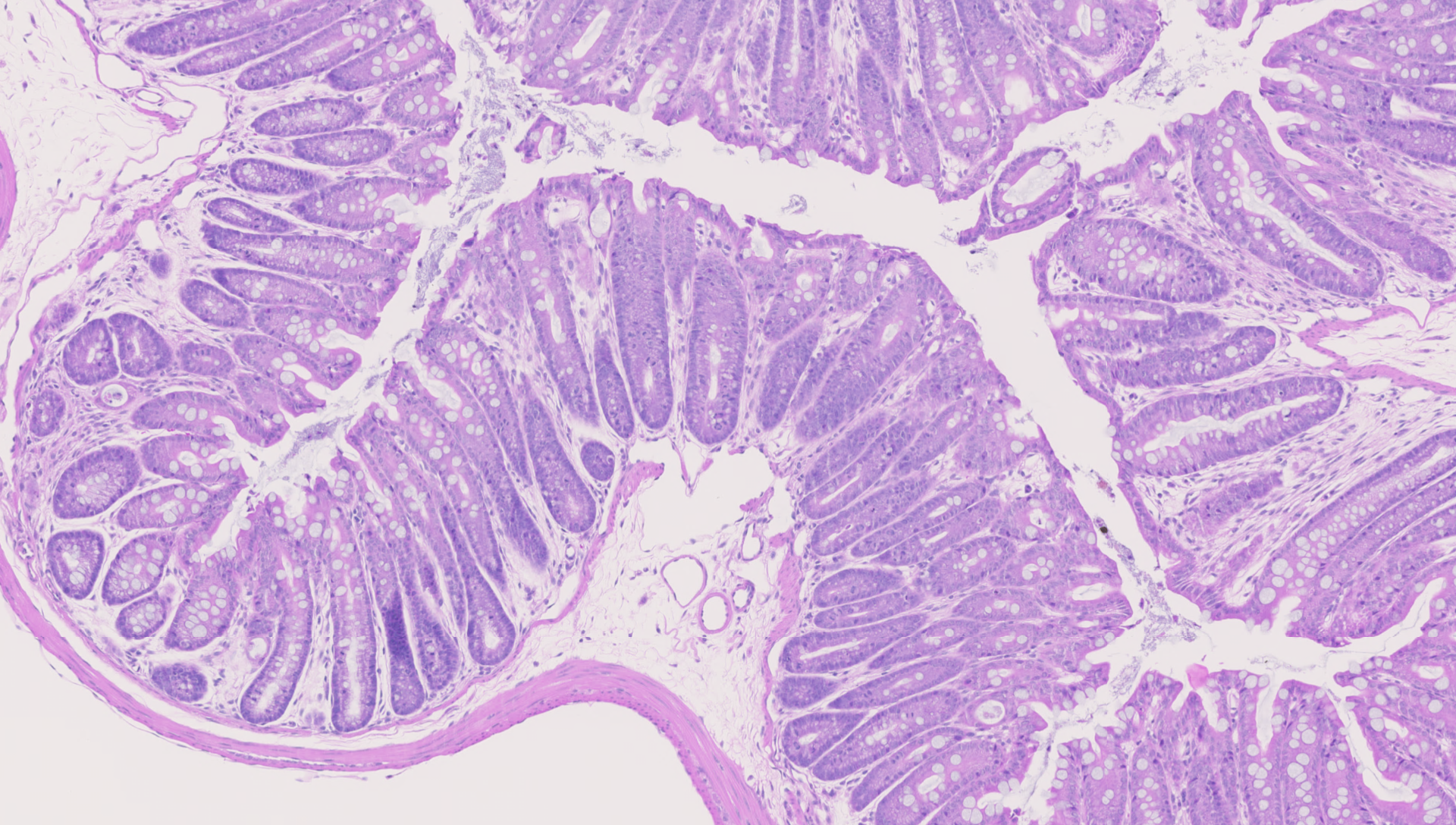

Supplement: Supplementary file 3 — Source data Fig. 1 [file 44321_2025_250_MOESM3_ESM.zip › Figure 1/1D/1D_ColonKO3_5X.tif]

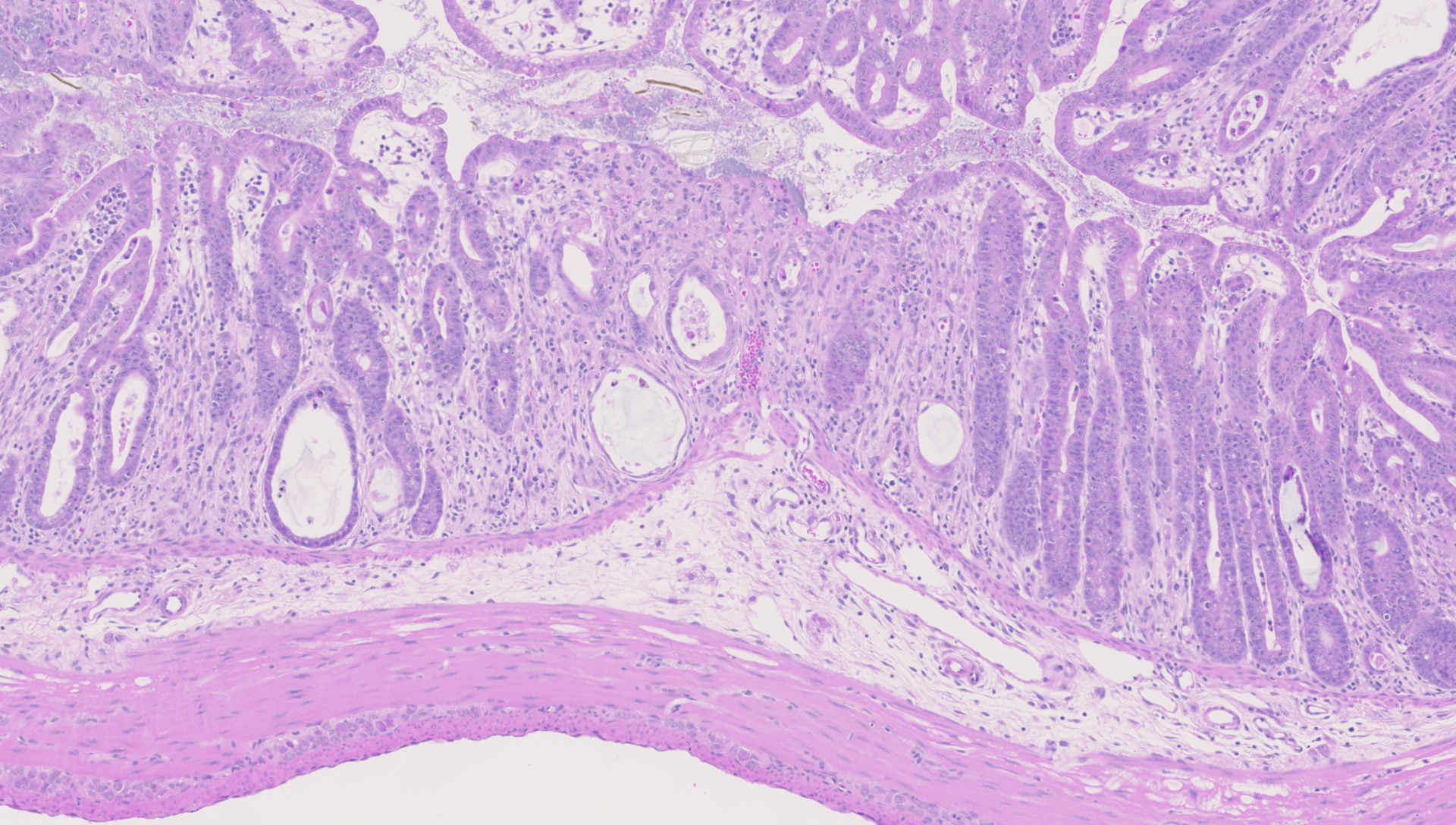

Supplement: Supplementary file 3 — Source data Fig. 1 [file 44321_2025_250_MOESM3_ESM.zip › Figure 1/1D/1D_ColonWT6_5X.tif]

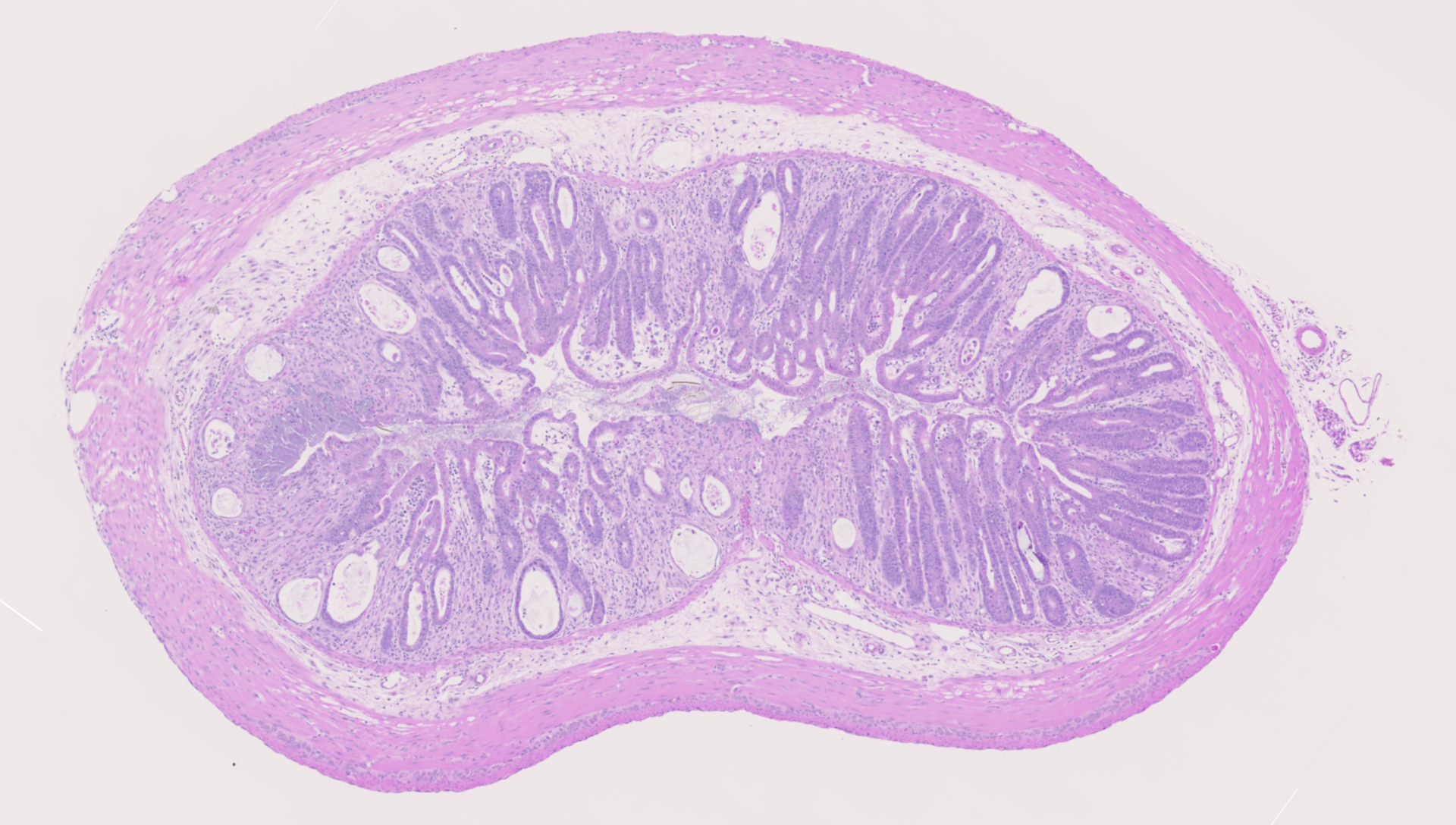

Supplement: Supplementary file 3 — Source data Fig. 1 [file 44321_2025_250_MOESM3_ESM.zip › Figure 1/1D/1D_ColonWT6_2.5X.tif]

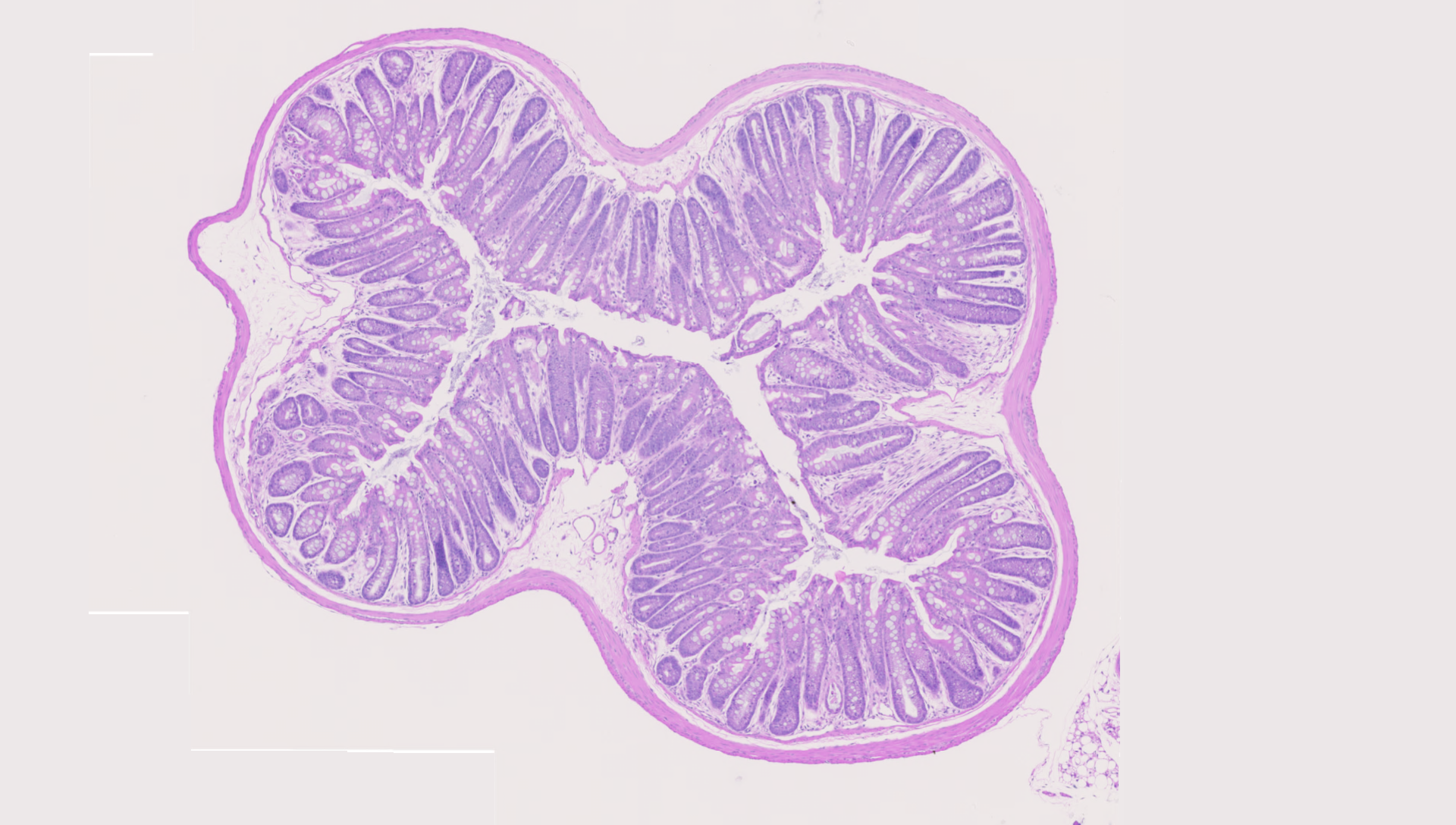

Supplement: Supplementary file 3 — Source data Fig. 1 [file 44321_2025_250_MOESM3_ESM.zip › Figure 1/1D/1D_ColonKO3_2.5X.tif]

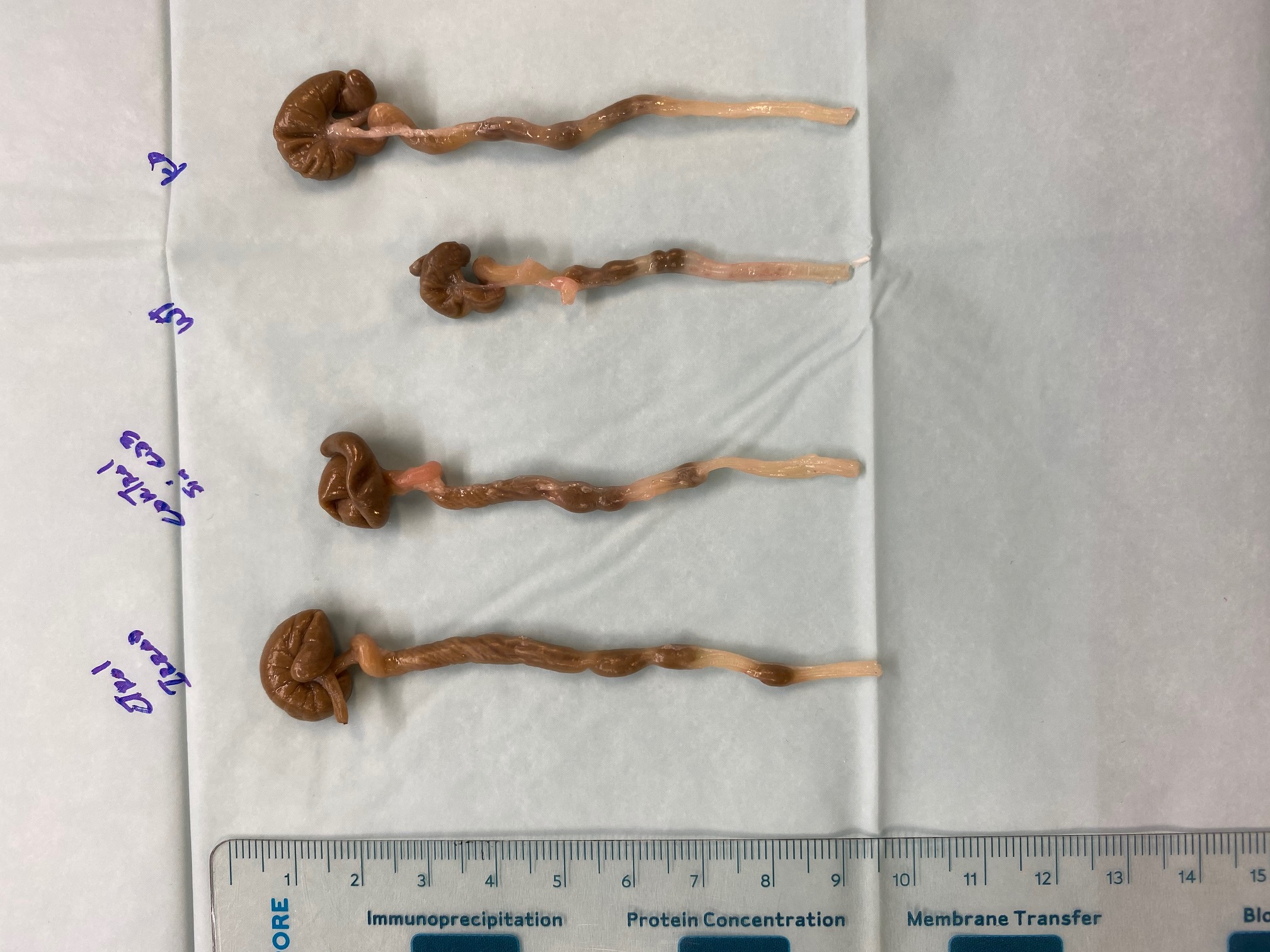

Supplement: Supplementary file 3 — Source data Fig. 1 [file 44321_2025_250_MOESM3_ESM.zip › Figure 1/1C/IMG_5459_RAWIMAGE.jpg]

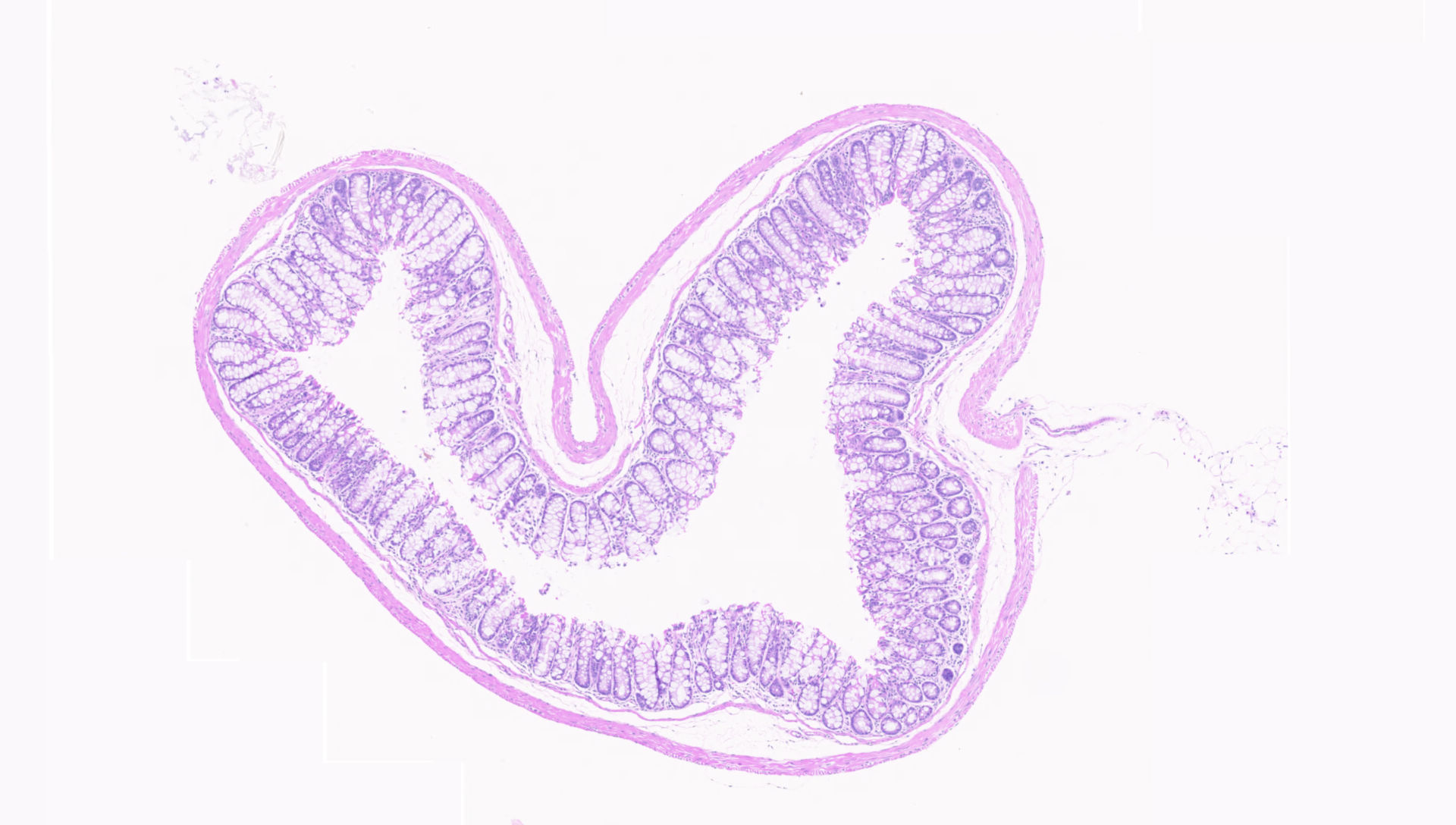

Supplement: Supplementary file 3 — Source data Fig. 1 [file 44321_2025_250_MOESM3_ESM.zip › Figure 1/1E/1E_ColonKO2_2.5x.jpg]

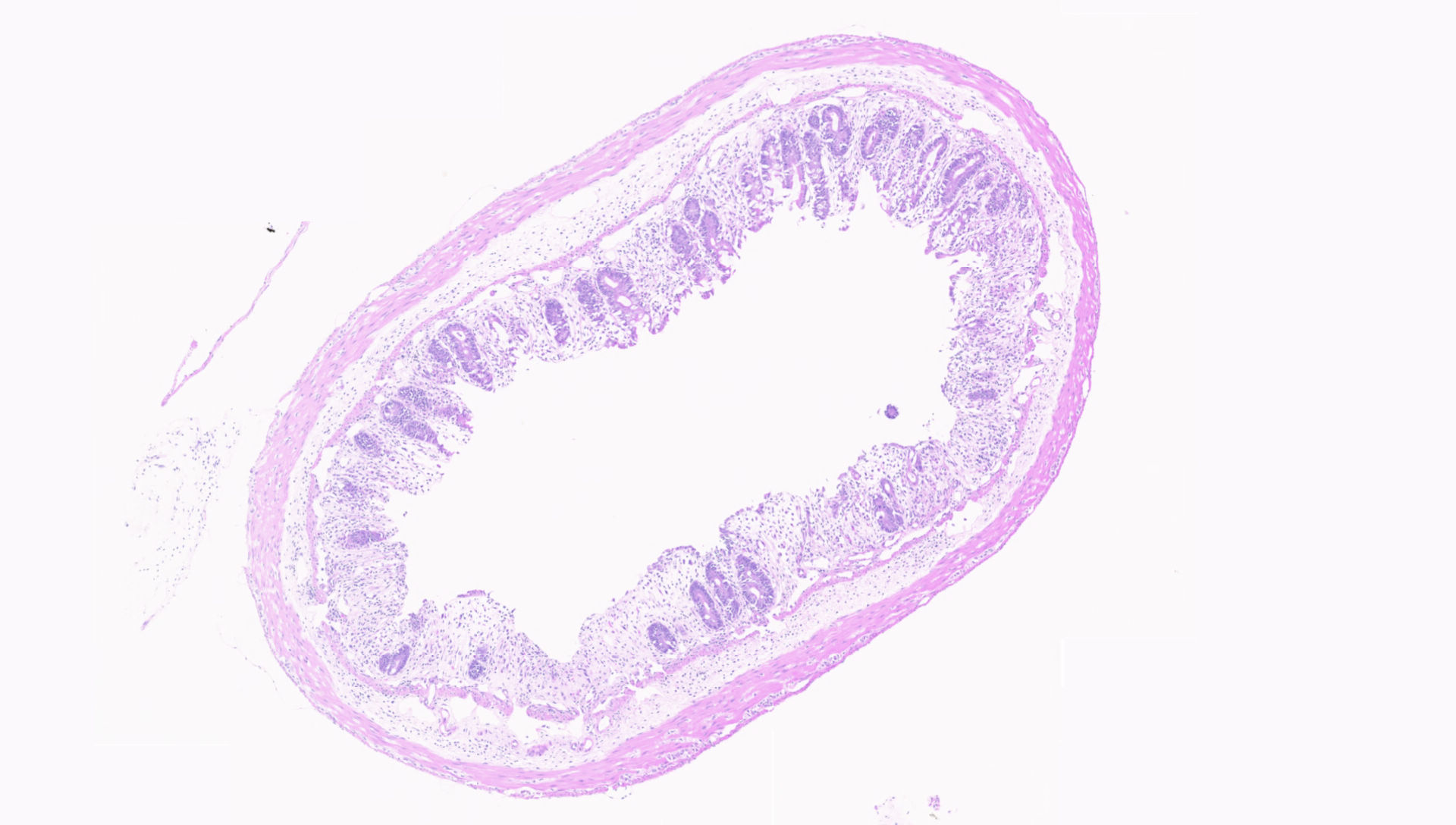

Supplement: Supplementary file 3 — Source data Fig. 1 [file 44321_2025_250_MOESM3_ESM.zip › Figure 1/1E/1E_ColonWT1_2.5x.jpg]

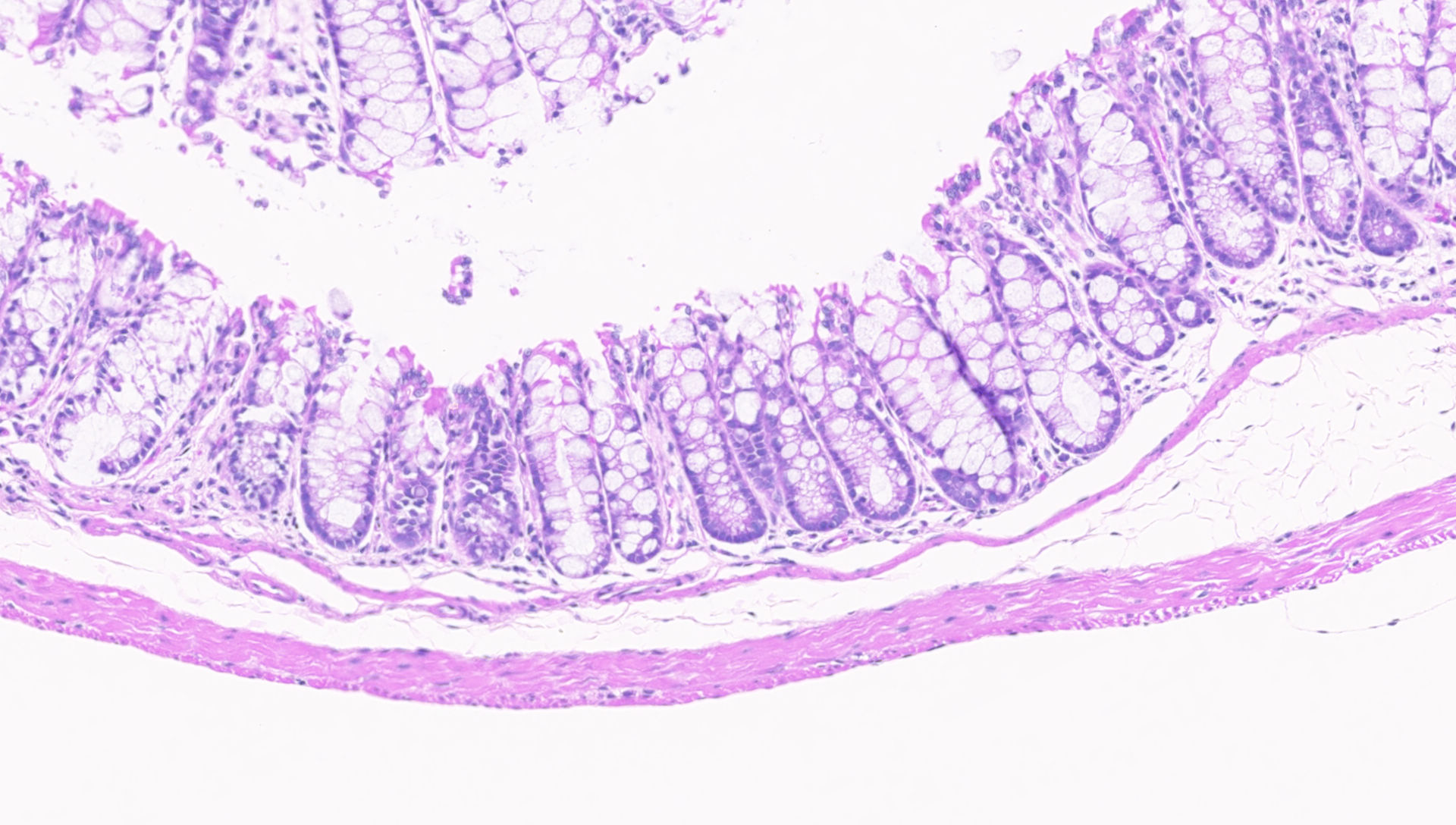

Supplement: Supplementary file 3 — Source data Fig. 1 [file 44321_2025_250_MOESM3_ESM.zip › Figure 1/1E/1E_ColonKO2_10x.jpg]

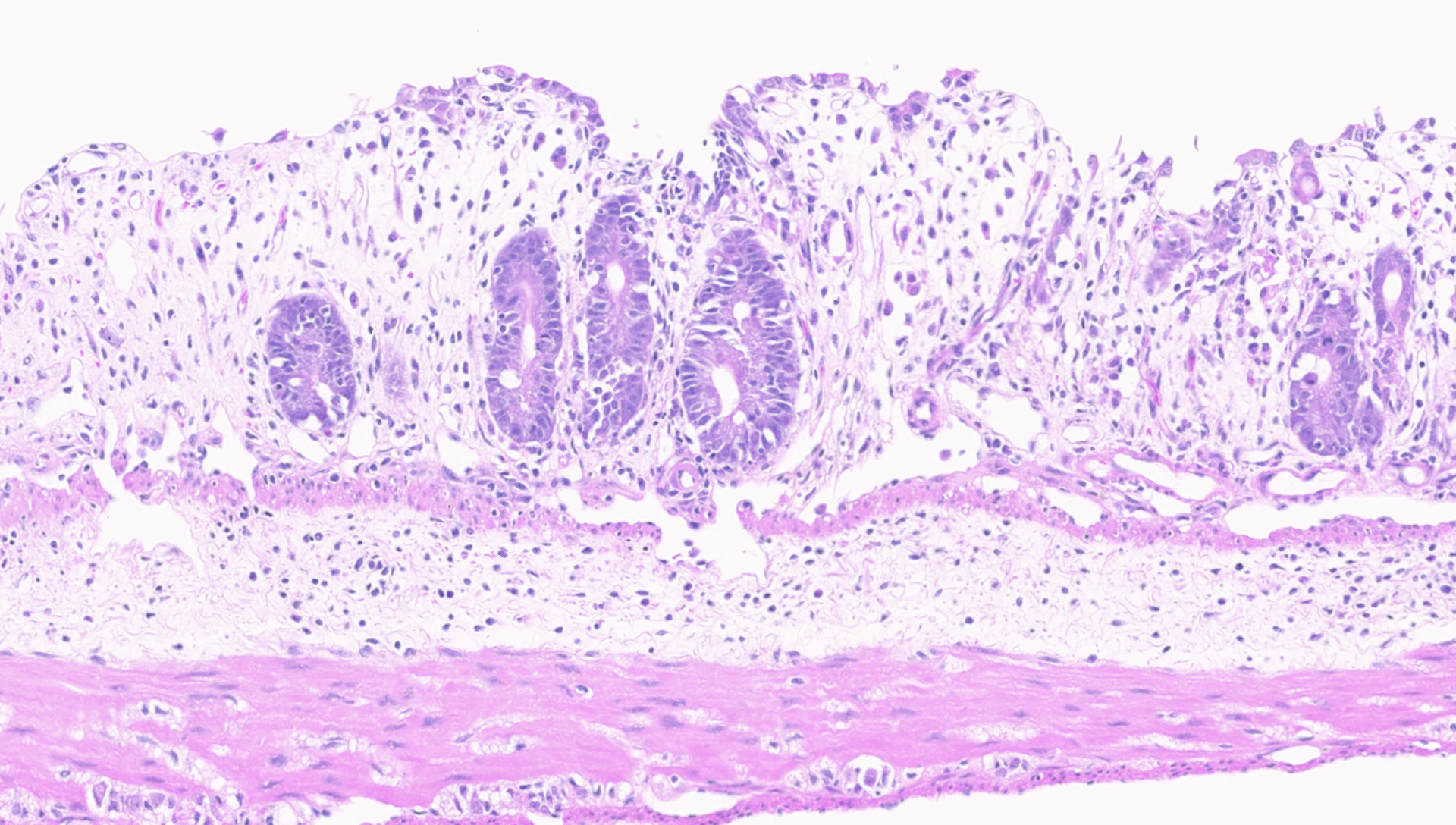

Supplement: Supplementary file 3 — Source data Fig. 1 [file 44321_2025_250_MOESM3_ESM.zip › Figure 1/1E/Fig1E_ColonWT1_10x.jpg]

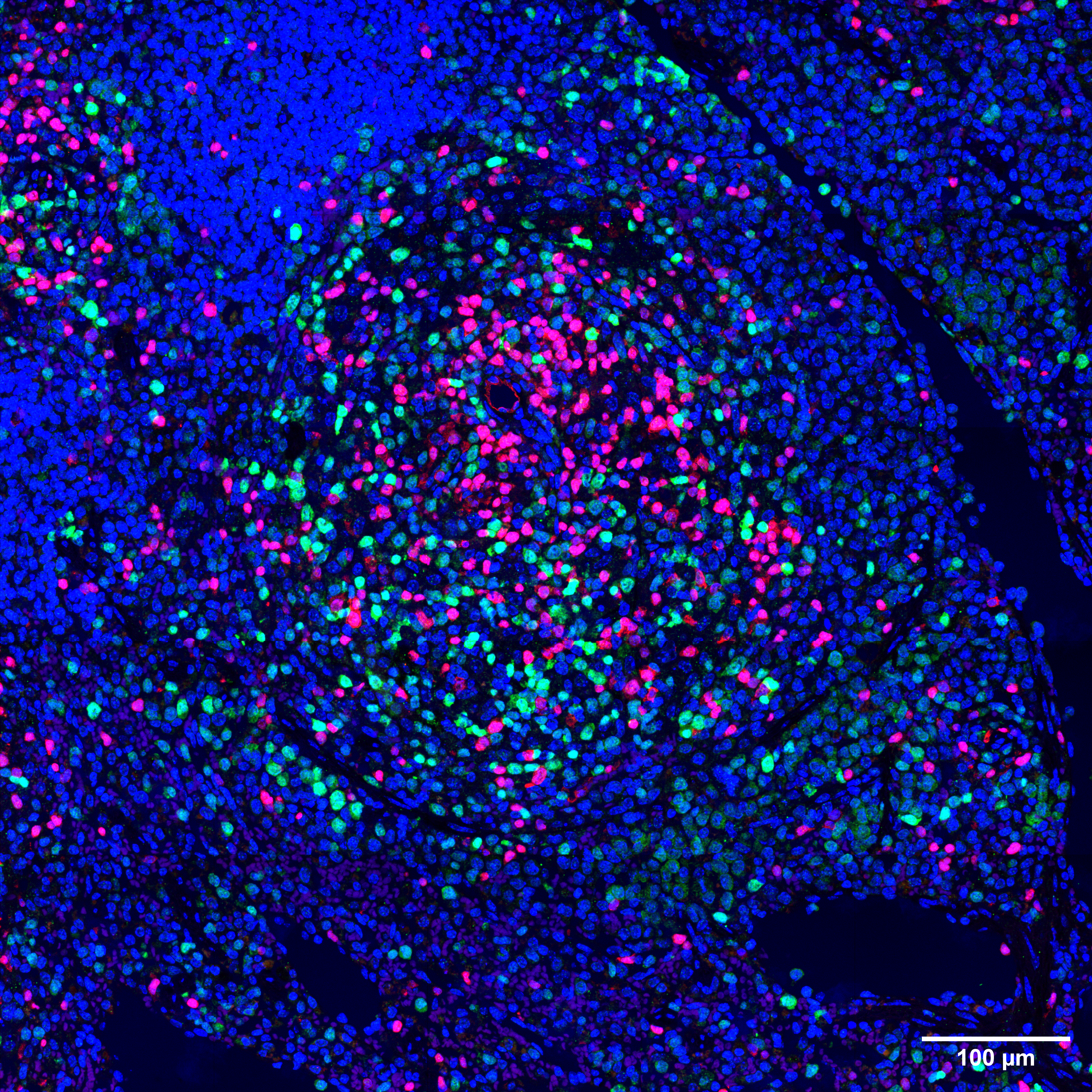

Supplement: Supplementary file 3 — Source data Fig. 1 [file 44321_2025_250_MOESM3_ESM.zip › Figure 1/1I/1I_Spleen_Composite-KO.tif]

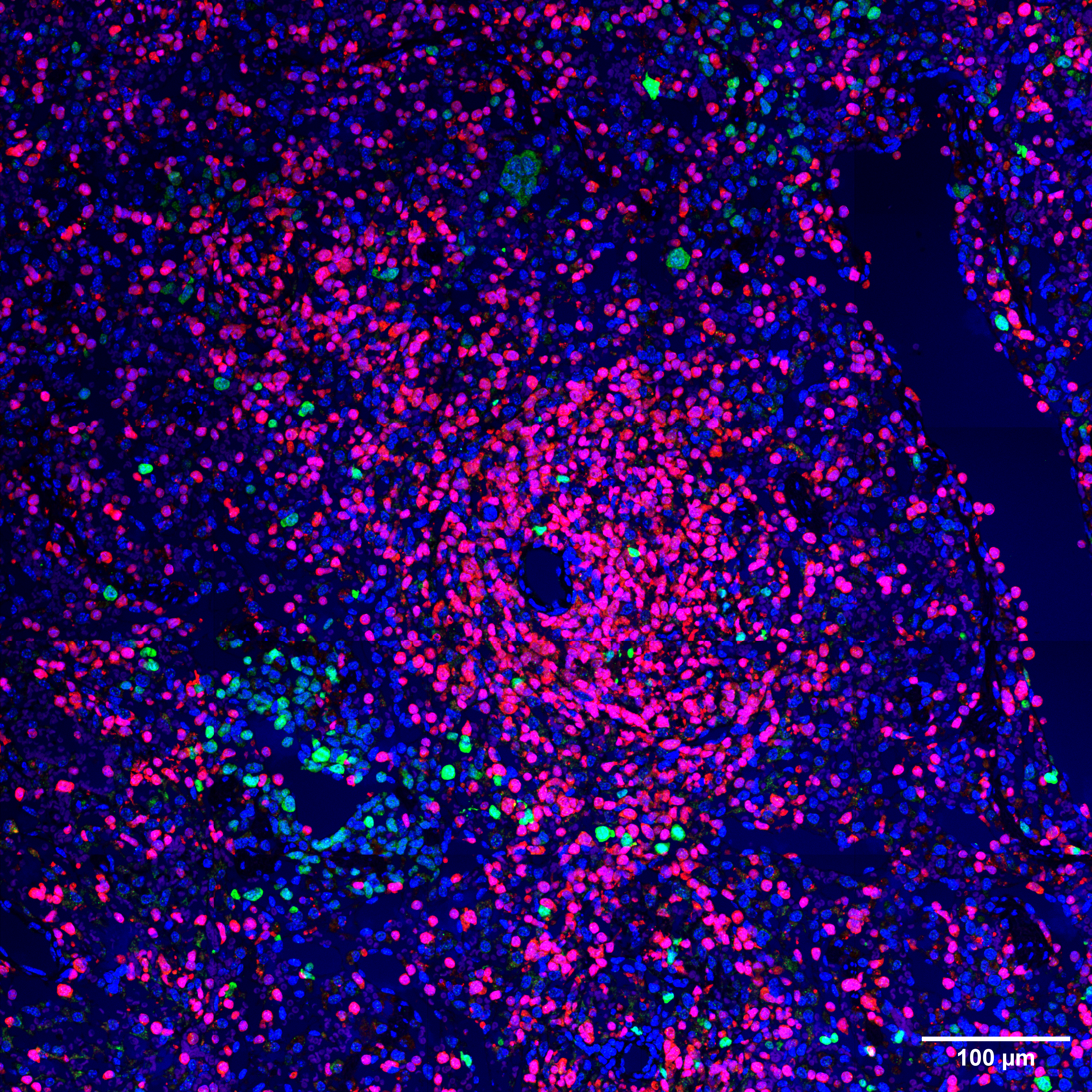

Supplement: Supplementary file 3 — Source data Fig. 1 [file 44321_2025_250_MOESM3_ESM.zip › Figure 1/1I/1I_Spleen_Composite-WT.tif]

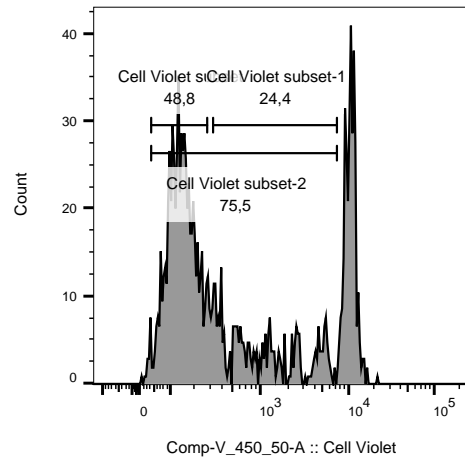

Proliferacion\_WT2.fcs  
tmt0  
1380

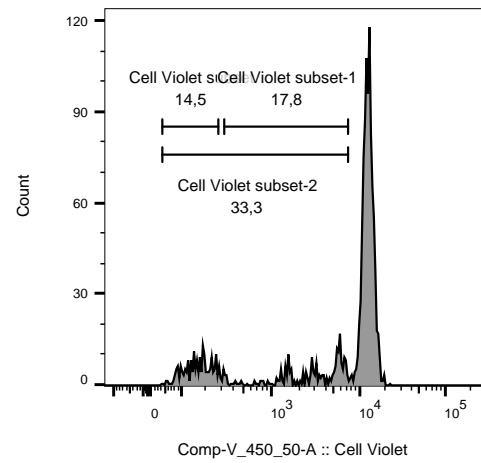

Proliferacion\_KO4.fcs  
tmt0  
1445

Supplement: Supplementary file 5 — Source data Fig. 3 [file 44321_2025_250_MOESM5_ESM.zip › Figure 3/3E/Plots FJ.pdf]
