# Supplementary material for: Restricting SLC7A5-mediated Leucine uptake in T cells prevents acute GVHD and maintains GVT response
Source: EMBO Mol Med. 2025 May 21;17(7):1631–65. doi: 10.1038/s44321-025-00250-2 (PMC12254332; doi:10.1038/s44321-025-00250-2)
Supplement: Supplementary file 11 — Expanded View Figures [file 44321_2025_250_MOESM11_ESM.pdf]

Expanded View Figures

Figure EV1. Effect of SLC7A5 genetic deletion in T cells in aGVHD model.

(**A**) Workflow for aGVHD model. STC: splenic T cell; TCDBM: T-cell-depleted bone marrow; Tmt: Tomato. (**B**) Representative images of the macroscopic appearance of $SLC7A5^{WT}$ → BALB/c and $SLC7A5^{\Delta CD4}$ → BALB/c mice at 40 d after transplant. (**C**) Representative density plots of $Ly6C^+$ (monocytes) and $Ly6C^+Ly6G^+$ (neutrophils) populations gated on $CD11b^+$ (myeloid) in colon of $SLC7A5^{WT}$ → BALB/c and $SLC7A5^{\Delta CD4}$ → BALB/c mice at 7 d post-transplant. (**D**) Representative density plots of total $CD4^+$ and $CD8^+$ populations gated on $CD3^+$ cells (left) and density plots of GFP vs Tmt gated on $CD4^+$ (middle) and $CD8^+$ (right) T cells in mesenteric lymph nodes (mLN) of $SLC7A5^{WT}$ → BALB/c and $SLC7A5^{\Delta CD4}$ → BALB/c mice at 7 d post-transplant. (**E**) Total count (left), GFP^+ (donor BM origin, middle) and GFP^-Tmt^- (host origin, right) of $CD4^+$ (top) and $CD8^+$ (bottom) cells in mLN and spleen of $SLC7A5^{WT}$ → BALB/c and $SLC7A5^{\Delta CD4}$ → BALB/c mice at 7 d post-transplant. A representative experiment of three is shown. $N = 4$–5 per group, mean ± SD, two-tailed Mann–Whitney's test, ns: not significant.

 © The Author(s)

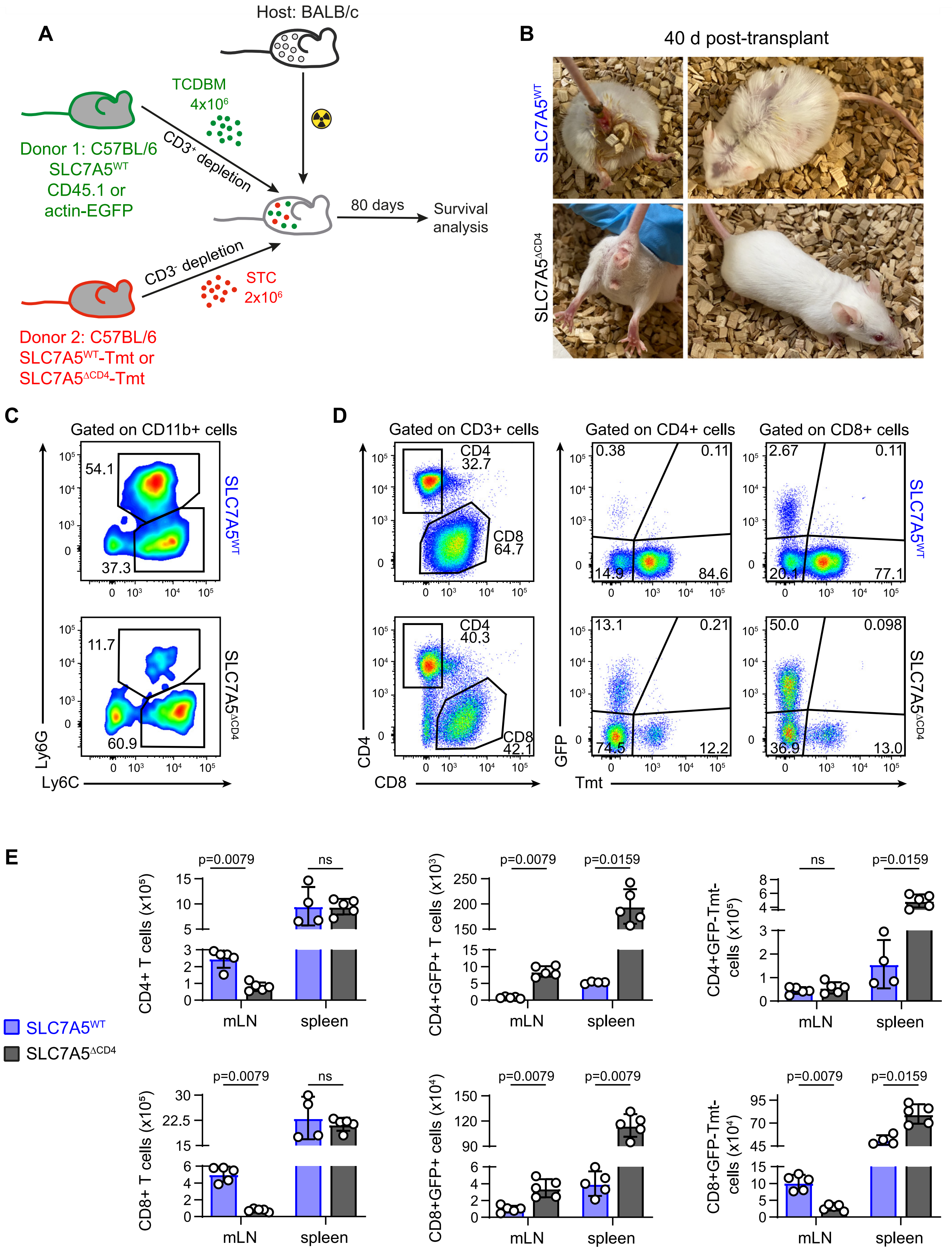
A
Host: BALB/c
TCDBM
4x10^6
CD3+ depletion
Donor 1: C57BL/6
SLC7A5WT
CD45.1 or
actin-EGFP
80 days
Survival analysis
CD3- depletion
STC
2x10^6
Donor 2: C57BL/6
SLC7A5WT-Tmt or
SLC7A5ΔCD4-Tmt
B
40 d post-transplant
SLC7A5WT
SLC7A5ΔCD4
C
Gated on CD11b+ cells
Ly6G
Ly6C
D
Gated on CD3+ cells
Gated on CD4+ cells
Gated on CD8+ cells
CD4
CD8
GFP
Tmt
E
SLC7A5WT
SLC7A5ΔCD4
CD4+ T cells (x10^5)
CD4+GFP+ T cells (x10^3)
CD4+GFP-Tmt- cells (x10^5)
CD8+ T cells (x10^5)
CD8+GFP+ cells (x10^4)
CD8+GFP-Tmt- cells (x10^4)
mLN
spleen

© The Author(s)

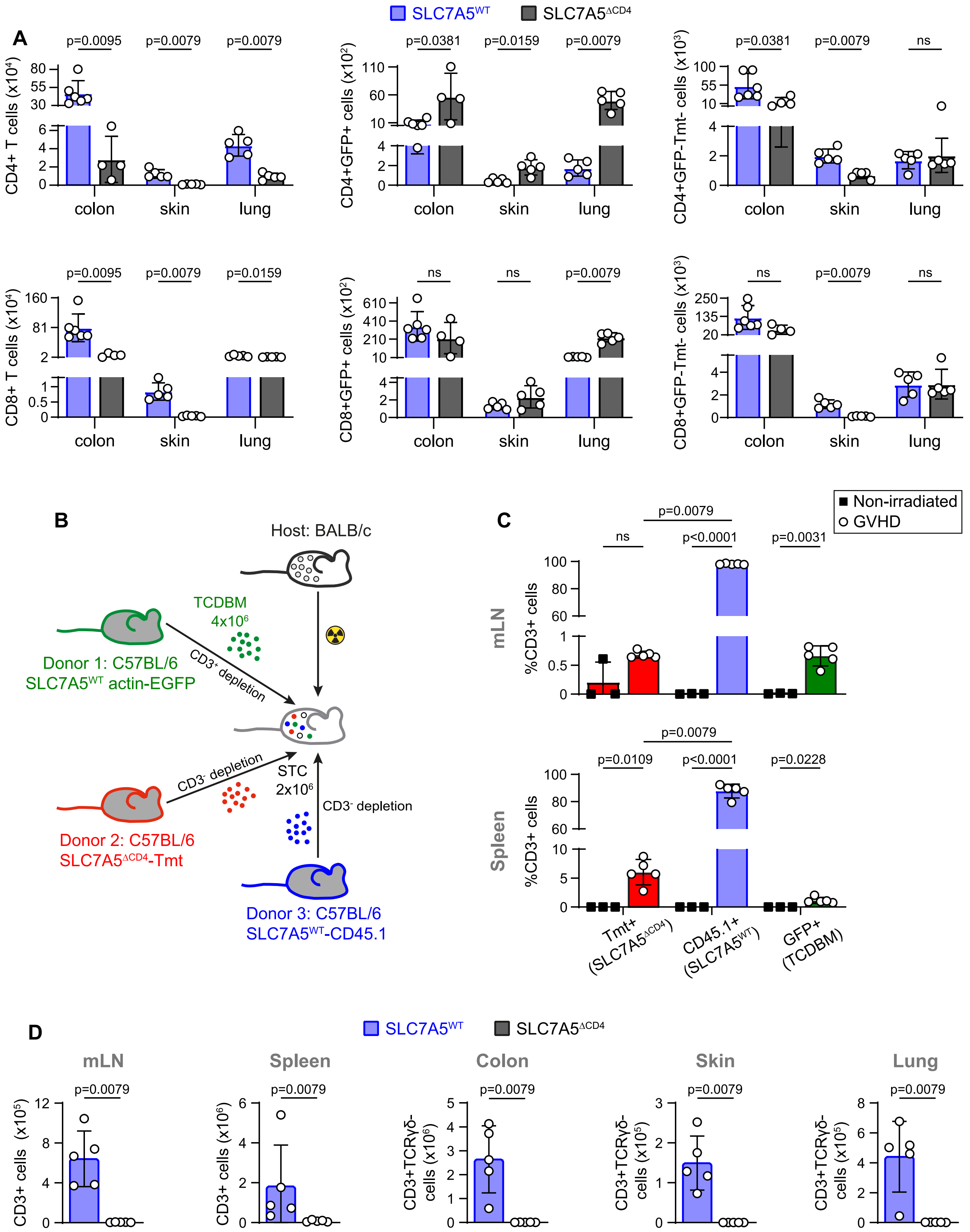
SLC7A5WT
SLC7A5ΔCD4
A
p=0.0095
p=0.0079
p=0.0079
CD4+ T cells (x10⁴)
colon
skin
lung
p=0.0381
p=0.0159
p=0.0079
CD4+GFP+ cells (x10²)
p=0.0381
p=0.0079
ns
CD4+GFP-Tmt- cells (x10³)
p=0.0095
p=0.0079
p=0.0159
CD8+ T cells (x10⁴)
ns
ns
p=0.0079
CD8+GFP+ cells (x10²)
ns
p=0.0079
ns
CD8+GFP-Tmt- cells (x10³)
B
Host: BALB/c
TCDBM
4x10⁶
CD3⁺ depletion
Donor 1: C57BL/6
SLC7A5WT actin-EGFP
CD3⁻ depletion
STC
2x10⁶
CD3⁻ depletion
Donor 2: C57BL/6
SLC7A5ΔCD4-Tmt
Donor 3: C57BL/6
SLC7A5WT-CD45.1
C
Non-irradiated
GVHD
mLN
%CD3+ cells
p=0.0079
ns
p<0.0001
p=0.0031
Spleen
p=0.0079
p=0.0109
p<0.0001
p=0.0228
Tmt+
(SLC7A5ΔCD4)
CD45.1+
(SLC7A5WT)
GFP+
(TCDBM)
D
SLC7A5WT
SLC7A5ΔCD4
mLN
p=0.0079
CD3+ cells (x10⁵)
Spleen
p=0.0079
CD3+ cells (x10⁶)
Colon
p=0.0079
CD3+TCRγδ- cells (x10⁶)
Skin
p=0.0079
CD3+TCRγδ- cells (x10⁵)
Lung
p=0.0079
CD3+TCRγδ- cells (x10⁵)

 © The Author(s)

Figure EV2. Role of SLC7A5 in T cell expansion and migration in aGVHD model.

(**A**) Total count (left), GFP^{+} (donor BM origin, middle) and $GFP^{-}Tmt^{-}$ (host origin, right) of $CD4^{+}$ (top) and $CD8^{+}$ (bottom) cells in colon, skin and lung of $SLC7A5^{WT} \rightarrow$ BALB/c and $SLC7A5^{\Delta CD4} \rightarrow$ BALB/c mice at 7 d post-transplant. (**B**) Workflow for competitive migration assay. STC: splenic T cell; TCDBM: T-cell-depleted bone marrow; Tmt: Tomato. (**C**) Percentages of $CD3^{+}$ cells expressing Tmt^{+} (STC-derived from $SLC7A5^{\Delta CD4}$ mice donor, red), $CD45.1^{+}$ (STC-derived from $SLC7A5^{WT}$ B6.SJL mice donor, blue), or GFP^{+} (TCDBM-derived from β-actin-eGFP mice donor, green) in mLN and spleen from non-irradiated (control) and irradiated (GVHD) BALB/c recipient mice in competitive migration assay. $N = 3$–5 per group, mean ± SD, Kruskal–Wallis test with Dunn's post-test for control and GVHD comparison or two-tailed Mann–Whitney's test for Tmt^{+} and $CD45.1^{+}$ of GVHD group comparison. (**D**) Absolute numbers of CD3 T cells $SLC7A5^{WT}$-$CD45.1^{+}$ and $SLC7A5^{\Delta CD4}$-Tmt^{+} in different tissues of GVHD mice of the competitive migration assay. A, D: $N = 4$–6 per group, mean ± SD, two-tailed Mann–Whitney's test. STC splenic T cell, TCDBM T-cell-depleted bone marrow, Tmt Tomato.

© The Author(s)

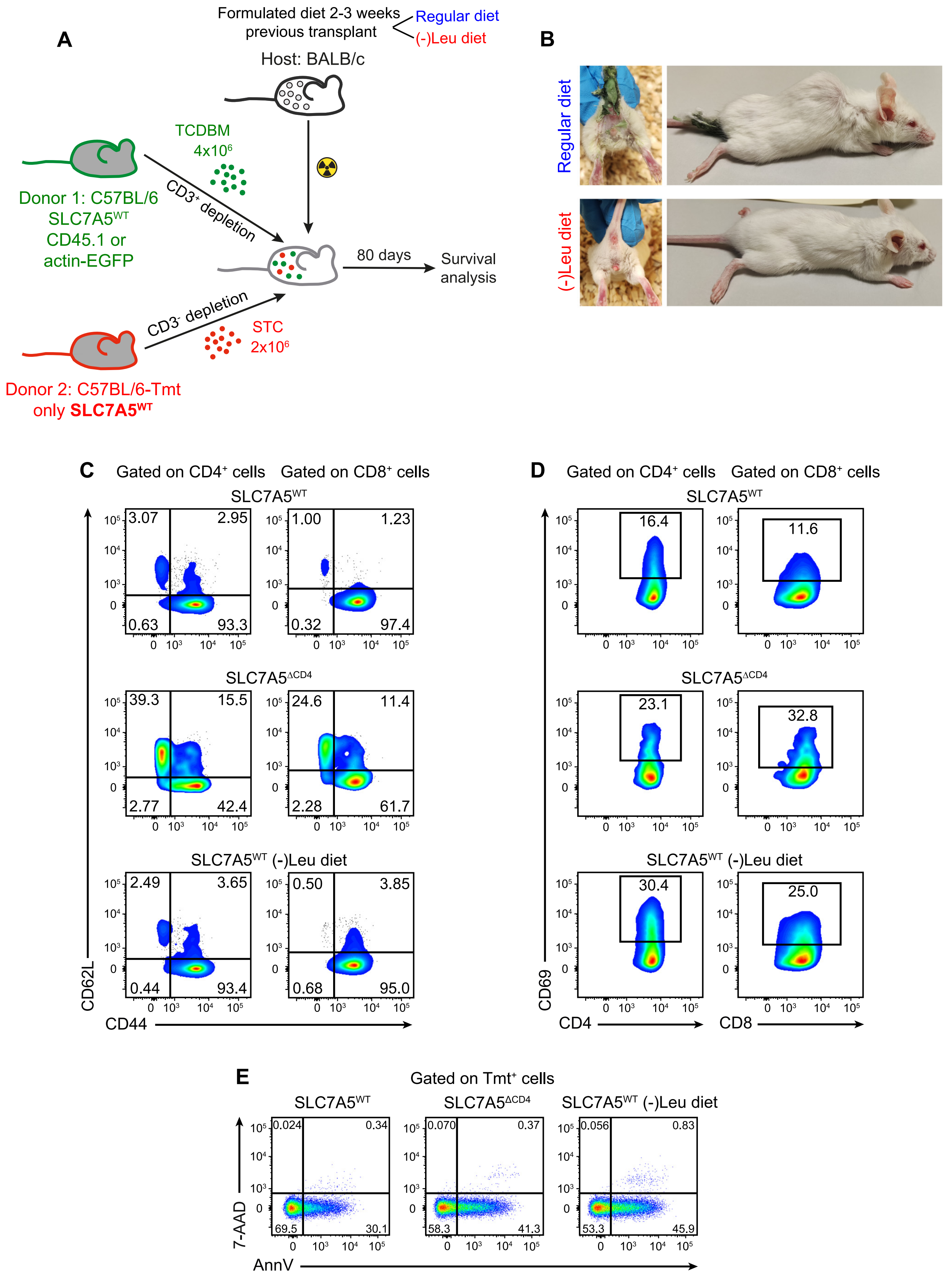
A
Formulated diet 2-3 weeks previous transplant
Regular diet
(-)Leu diet
Host: BALB/c
TCDBM 4x10^6
Donor 1: C57BL/6 SLC7A5^WT CD45.1 or actin-EGFP
CD3+ depletion
80 days
Survival analysis
CD3- depletion
STC 2x10^6
Donor 2: C57BL/6-Tmt only SLC7A5^WT
B
Regular diet
(-)Leu diet
C
Gated on CD4+ cells
Gated on CD8+ cells
SLC7A5^WT
3.07
2.95
0.63
93.3
1.00
1.23
0.32
97.4
SLC7A5^ΔCD4
39.3
15.5
2.77
42.4
24.6
11.4
2.28
61.7
SLC7A5^WT (-)Leu diet
2.49
3.65
0.44
93.4
0.50
3.85
0.68
95.0
CD62L
CD44
D
Gated on CD4+ cells
Gated on CD8+ cells
SLC7A5^WT
16.4
11.6
SLC7A5^ΔCD4
23.1
32.8
SLC7A5^WT (-)Leu diet
30.4
25.0
CD69
CD4
CD8
E
Gated on Tmt+ cells
SLC7A5^WT
SLC7A5^ΔCD4
SLC7A5^WT (-)Leu diet
0.024
0.34
69.5
30.1
0.070
0.37
58.3
41.3
0.056
0.83
53.3
45.9
7-AAD
AnnV

© The Author(s)

Figure EV3. Impact of dietary L-Leu restriction on T cell activation and survival in aGVHD model.

(**A**) Workflow for the aGVHD model with dietary restriction of L-leucine. STC splenic T cell, TCDBM T-cell-depleted bone marrow, Tmt Tomato. (**B**) Representative images of the macroscopic appearance of SLC7A5^{WT} → BALB/c mice fed with regular or (-)Leu diet at 57 d post-transplant. (**C**) Representative density plots of CD44 vs CD62L gated on allogenic CD4^{+} and CD8^{+} cells of spleen from (-)Leu diet-fed SLC7A5^{WT} → BALB/c mice and regular-diet fed BALB/c host mice simultaneously injected with SLC7A5$^{\Delta CD4}$ and SLC7A5^{WT} T cells at 7 d post-transplant. (**D**) Representative density plots of CD69 in allogenic CD4^{+} (left) and CD8^{+} (right) cells of spleen from mice described in **(C)**. (**E**) Representative density plots of AnnV vs 7-AAD gated on total Tmt^{+} cells of mesenteric lymph nodes from SLC7A5^{WT} → BALB/c mice fed with regular or (-)Leu diet and SLC7A5$^{\Delta CD4}$ → BALB/c mice at 7 d post-transplant.

© The Author(s)

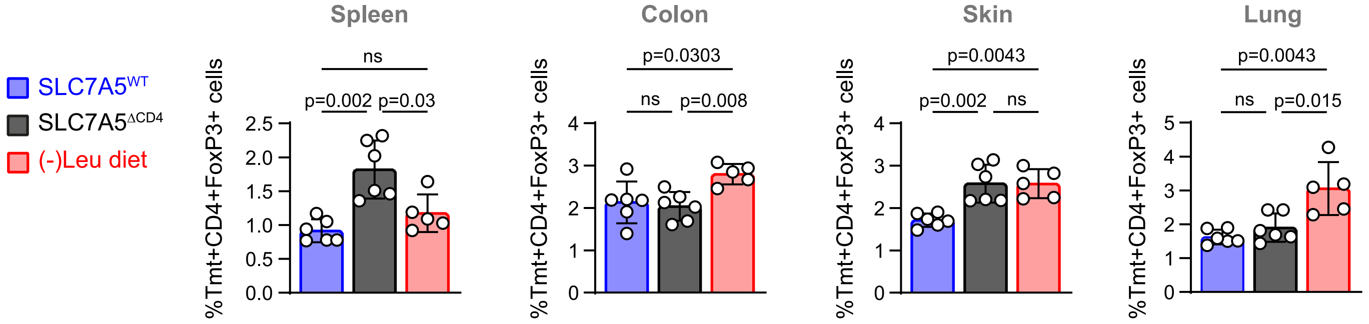

Figure EV4. Frequency of regulatory T cells.

Frequencies of FoxP3 cells gated on Tmt$^+$CD4$^+$ of different tissues from SLC7A5^{WT} → BALB/c mice fed with regular or (-)Leu diet and SLC7A5$^{\Delta CD4}$ → BALB/c mice at 7 d post-transplant. N = 5–6 per group, mean ± SD, two-tailed Mann–Whitney's test, ns: not significant.

 © The Author(s)

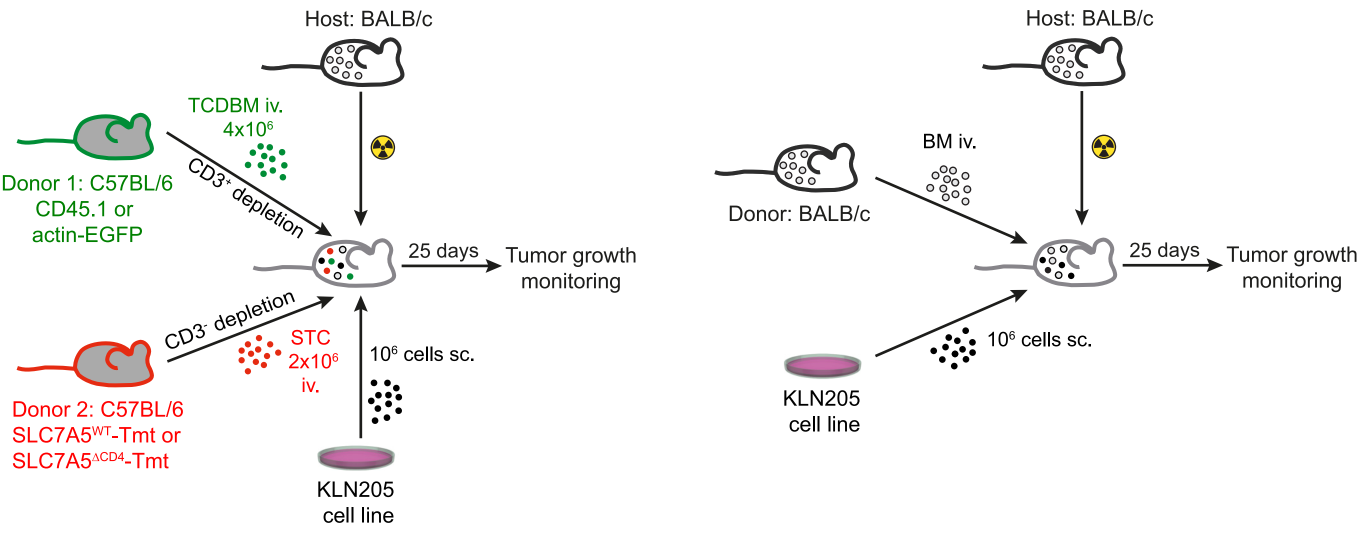

Figure EV5. Experimental design to evaluate GVT response.

Workflow for GVT model comparing the effect of $SLC7A5^{WT}$ and $SLC7A5^{\Delta CD4}$ T cells in GVHD groups (left) and control mice (right). STC splenic T cell, TCDBM T-cell-depleted bone marrow, Tmt Tomato.

© The Author(s)

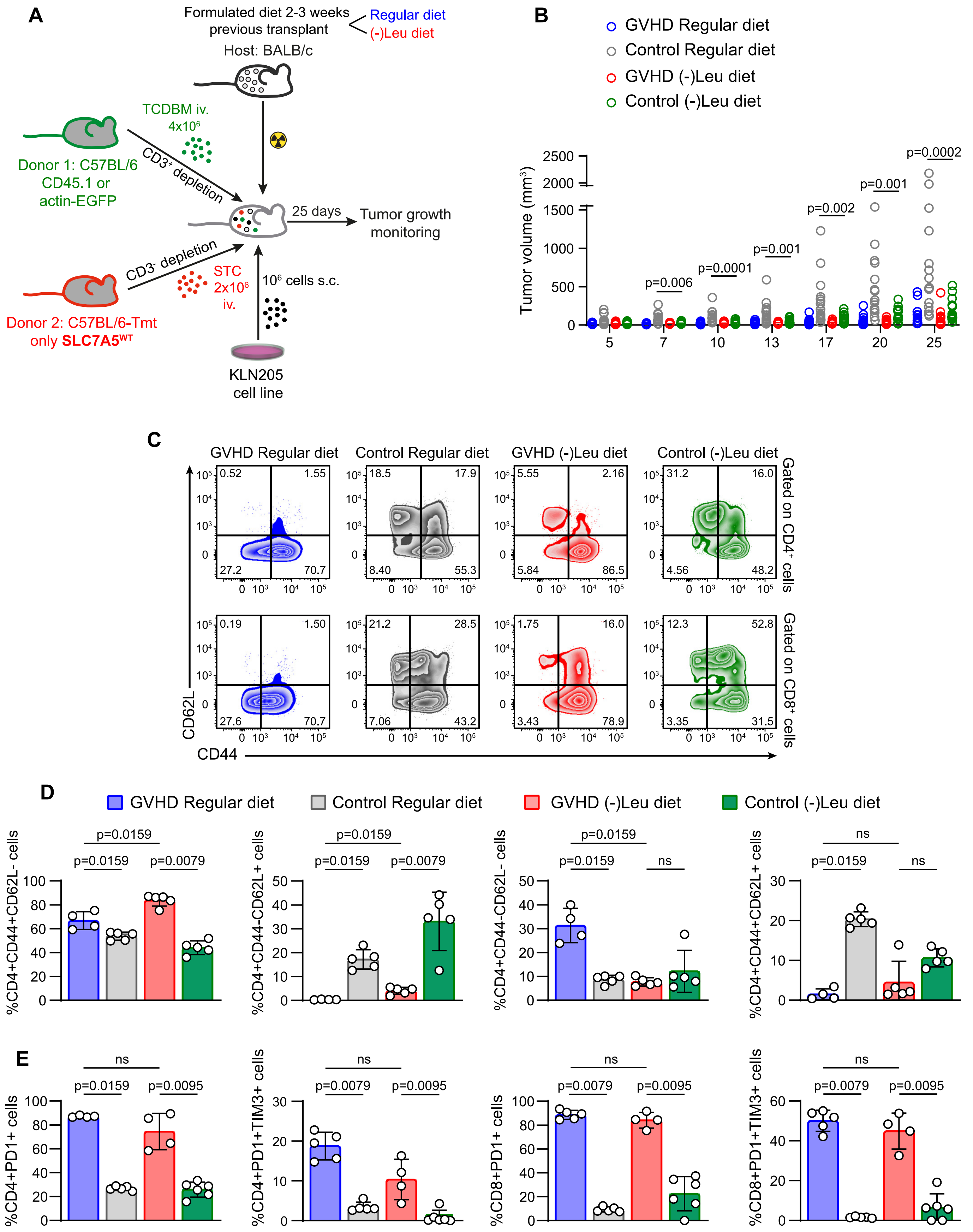
A
Formulated diet 2-3 weeks previous transplant
Regular diet
(-)Leu diet
Host: BALB/c
TCDBM iv. 4x10^6
CD3+ depletion
Donor 1: C57BL/6 CD45.1 or actin-EGFP
25 days
Tumor growth monitoring
CD3- depletion
STC 2x10^6 iv.
10^6 cells s.c.
Donor 2: C57BL/6-Tmt only SLC7A5WT
KLN205 cell line
B
GVHD Regular diet
Control Regular diet
GVHD (-)Leu diet
Control (-)Leu diet
Tumor volume (mm3)
p=0.006
p=0.0001
p=0.001
p=0.002
p=0.001
p=0.0002
C
GVHD Regular diet
Control Regular diet
GVHD (-)Leu diet
Control (-)Leu diet
Gated on CD4+ cells
Gated on CD8+ cells
CD62L
CD44
D
%CD4+CD44+CD62L- cells
%CD4+CD44-CD62L+ cells
%CD4+CD44-CD62L- cells
%CD4+CD44+CD62L+ cells
E
%CD4+PD1+ cells
%CD4+PD1+TIM3+ cells
%CD8+PD1+ cells
%CD8+PD1+TIM3+ cells

Figure EV6. Evaluation of dietary L-Leu intake in tumor growth and GVT response.

(**A**) Workflow for GVT model comparing the effect of control and (-)Leu diet. STC splenic T cell, TCDBM T-cell-depleted bone marrow, Tmt Tomato. (**B**) Volume of subcutaneous tumors measured with an electronic calliper. A pool of three independent experiments is shown. Only live animals at the end point are represented: control regular diet $N = 16$, control (-)Leu diet $N = 17$, GVHD regular diet $N = 14$, GVHD (-)Leu $N = 15$. All indicated p values are calculated from control regular diet versus control (-)Leu diet, two-tailed Mann–Whitney's test. (**C**, **D**) Representative density plots (**C**) and frequencies (**D**) of naive ($CD44^{-}CD62L^{+}$), effector memory ($CD44^{+}CD62L^{-}$), double negative ($CD44^{-}CD62L^{-}$), and central memory ($CD44^{+}CD62L^{+}$) T cells gated on spleen $Tmt^{+}CD4^{+}$ and $Tmt^{+}CD8^{+}$ cells in GVHD groups and $CD4^{+}$ and $CD8^{+}$ cells in control groups after 25 d of sc. tumor injection. (**E**) Frequencies of PD1 and TIM3 gated on $Tmt^{+}CD4^{+}CD44^{+}$ or $Tmt^{+}CD8^{+}CD44^{+}$ cells of spleen from GVHD groups and $CD4^{+}CD44^{+}$ or $CD8^{+}CD44^{+}$ cells of spleen from control groups after 25 d of sc. tumor injection. (**D**, **E**): $N = 4$–5 per group, mean ± SD, two-tailed Mann–Whitney's test.

© The Author(s)
